# The coupling relationship and driving mechanism between ecological environment and high-quality economic development in the Middle Yellow River Basin

**DOI:** 10.1038/s41598-025-94462-8

**Published:** 2025-03-28

**Authors:** Shuo Yang, Zhongwu Zhang, Jinyuan Zhang, Xue Bai, FanFan Hu, Kunwei Zhang, Shiyu Wang

**Affiliations:** 1 College of Geographic Science, Shanxi Normal University, Taiyuan, 030031 China; 2 Institute of Human Geography, Shanxi Normal University, Taiyuan, 030031 China; 3 School of Surveying and Land Information Engineering, Henan Polytechnic University, Jiaozuo, 454003 China

**Keywords:** Ecological environment, High-quality economic development, Coupling relationship, Driving mechanism, Middle Yellow River Basin

## Abstract

Promoting the dynamic balance between economic development and ecological environment is key to achieving the “dual carbon” goals and sustainable development. The Middle Yellow River Basin, characterized by severe soil erosion and intensive resource utilization, serve as a critical area for advancing ecological protection and high-quality development in the Yellow River Basin. This study examines the spatial–temporal differentiation and coupling coordination characteristics of the ecological environment (EE) and high-quality economic development (HQED) across 226 counties in the Middle Yellow River Basin from 2010 to 2020. Utilizing the Random Forest model and the Geographical and Temporal Weighted Regression model, the study investigates the driving mechanism of high-quality economic development on ecological environment. The zoning management strategy is proposed based on the types of coupling coordination and the dominant driving factors, with the aim of providing theoretical support for sustainable development in the river basins. The results show that: (1) During the study period, the level of ecological environment initially declined and then improved, while high-quality economic development consistently increased. The EE exhibited a spatial pattern of "southeast low, northwest high," while the distribution pattern of HQED was the reverse. (2) The coupling coordination degree considerably increased after 2015, displaying the spatial pattern characterized by higher levels in the southeast and northwest and lower levels in the central region, with the strong spatial positive correlation. (3) Forest cover rate, PM2.5 concentration, agricultural fertilizer application intensity, and market activity make high contributions to the ecological environment, making them key drivers. Forest cover rate is the strongest positive driver, while PM2.5 concentration is the strongest negative driver. There are evident spatial distribution differences among the various driving factors. Ultimately, the study area is divided into six types of zones, and corresponding development strategies is proposed.

## Introduction

The human–environment relationship territorial system is a fundamental area of geographic research, where human development’s exploitation and utilization of resources and the environment, along with their responses, represent the concrete manifestation of this relationship^1^. Under the intensifying global climate change and expanding human activities, the conflict between ecological conservation and economic development has become increasingly prominent, emerging as a critical constraint to sustainable development^2,3^. Currently, the United Nations 2030 Sustainable Development Agenda (SDGs) has entered a critical phase. China’s implementation of the high-quality development strategy provides a new approach to coordinating the relationship between the economy and ecology^4^. Objectively assessing the relationship between ecological environment and high-quality economic development provides not only scientific pathways for achieving SDG 8 (Economic Growth) and SDG 15 (Life on Land), but also theoretical support for guiding regional development directions.

The ecological environment (EE) and high-quality economic development (HQED) are two interrelated complex systems. The EE provides the material basis for HQED, with the most direct manifestation being ecosystem services. Ecosystem services (ES) are defined as the benefits people derive from ecosystems^5^. Ecosystem service value (ESV) is the monetary value of ecosystem services and is an important representation of the ecological environment, reflecting the interaction between regional economic development and the ecological environment^6^. The value of ecosystem services is systematically revealed through functional classification and dynamic quantification, thereby providing a comprehensive understanding of regional ecological environment conditions^7^. Since Costanza et al.^8^ quantified global ESV, scholars have extensively applied this framework across multiple domains, such as analyzing trade-offs and synergies^9^, ecological compensation^10^, and land-use planning^11^. Most studies calculate a single equivalence factor and adjust it based on biomass^12^ and economic development levels^13^. However, the impact of biomass differences across regions and price fluctuations across years on ESV is often overlooked. Therefore, this study calculates biomass correction factors based on vegetation cover in different regions and eliminates the effects of inflation by using the Consumer Price Index (CPI), thus enabling a more accurate estimation of ESV. At the same time, HQED has the driving effect on the EE. As a new development model proposed by China, the concept of HQED continues to deepen and evolve. Research primarily focuses on measuring and evaluating the status of high-quality development from perspectives such as the new development philosophy^14^, theoretical logic^15^, and the concept of Chinese-style modernization^16^. However, studies exploring the relationship between HQED and EE from the perspective of main functional zoning are still in need of further expansion.

The harmonious evolution of the ecological environment and socio-economic development is an essential path to achieving sustainable development at both national and regional levels^17^. Research outcomes from scholars both domestically and internationally on the connotations of ecological protection and high-quality economic development, as well as their coupling and coordination relationships, continue to emerge. Studies have applied the Environmental Kuznets Curve (EKC)^18^ and the Pressure-State-Response (PSR)^19^ model to explore the coupling relationship between the ecological environment and economic development. In addition, numerous quantitative evaluation models have been developed, including methods such as comprehensive index evaluation^20^, coupling coordination degree model^21^, ecological footprint analysis^22^, energy value analysis^23^, and material flow analysis^24^. At different stages of regional development, the importance of the ecological environment and socio-economy varies according to the government’s planning positioning^25^. Therefore, it is necessary to incorporate government decision-making into the evaluation framework. This paper sets different weights based on the main functional zoning, which can better highlight the coupling relationship between EE and HQED. Moreover, current research scales involve national^26^, provincial^27^, urban agglomeration^28^, city^29^, and county levels^30^.

In terms of studying driving mechanisms, research has predominantly focused on the impact of external factors, with limited exploration into the internal mechanisms of both systems. Common methods for analyzing driving mechanisms include spatial data overlay^31^, the STIRPAT model^32^, system dynamics model^33^, the geographic detector model^34^, and geographically weighted regression model (GWR)^35^. In recent years, the random forest model (RF) has matured and been increasingly applied across various fields. Compared with traditional regression models, the random forest model offers high accuracy, reduces overfitting, manages high-dimensional data, and provides feature importance measures. Variable importance in random forest provides a precise measure of each factor’s contribution to the dependent variable^36^. The geographical and temporal weighted regression (GTWR) model extends GWR by introducing the temporal dimension, fully accounting for both spatial and temporal non-stationarity^37^. The RF model reflects the overall contribution of each factor to the dependent variable, whereas the GTWR model focuses on revealing the spatial heterogeneity in the response of the dependent variable to changes in factors. Therefore, this study employs both RF and GTWR models to provide more comprehensive understanding of the driving mechanism of high-quality economic development on the ecological environment.

With the elevation of ecological protection and high-quality development in the Yellow River Basin to the national strategy, achieving harmony between humans and nature has become particularly crucial^38^. The ecosystem stability in the Middle Yellow River Basin is poor, and the fragility of EE constrains economic development. Traditional modes of economic growth have led to severe ecological pollution, with phenomena such as the resource curse and path dependence becoming increasingly evident. Investigating the relationship between EE and HQED in the Middle Yellow River Basin can help deepen the understanding of regional sustainable development and human–environment interaction theories, and provide new perspectives and theoretical support for the coordinated governance and development of the region.

**Fig. 1:**
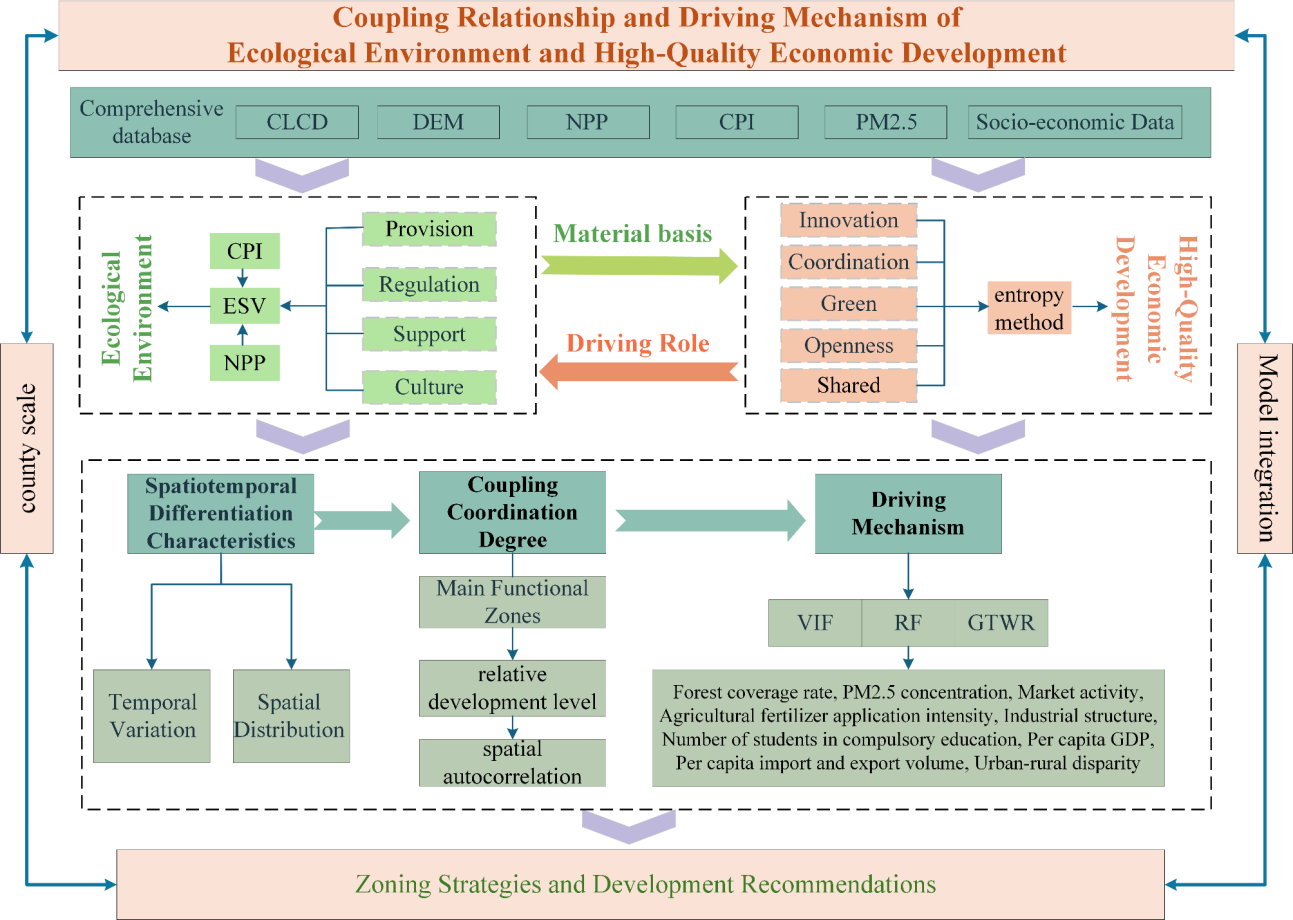
Evaluation framework for the spatiotemporal relationship of EE and HQED.

The objective of the synergistic evolution between ecological environment and socio-economic development is to achieve a state of harmonious coexistence between humans and nature. By studying the coupling coordination between the EE and HQED, it is possible to adjust the system structure and optimize its functions. The ecological environment is the material basis for high-quality economic development. Achieving ecological asset assessment aids in optimizing the intensive use of natural resources, maintaining ecological balance, and enhancing cultural and recreational benefits. The driving role of HQED is manifested in ecological engineering construction and the green transformation of industries, thereby improving overall efficiency and achieving energy conservation, emission reduction, and low-carbon development. Therefore, this study examines the 226 counties in the Middle Yellow River Basin, using ESV to represent EE and integrating the evaluation index system for HQED to analyze the coupling relationship between them. By applying the coupling coordination degree (CCD) models and spatial autocorrelation model, we assess the level of coupling coordination and spatial clustering characteristics between EE and HQED. Finally, the RF and GTWR models are used to reveal the driving mechanism of HQED on the EE, providing a new reference framework for policy-making in the Middle Yellow River Basin (Fig. 1). The primary objectives of the study are: (1) to assess the levels of EE and HQED, and to explore their spatiotemporal differentiation characteristics; (2) based on the main functional zones, to analyze the coupling relationship between the EE and HQED, as well as their spatial agglomeration characteristics; (3) to explore the driving factors of HQED on EE and their contribution degrees, and to study the spatiotemporal heterogeneity of each factor respectively. Furthermore, optimization strategies are proposed in accordance with different county-level coupling coordination types and dominant driving factors.

## Materials and methods

### Study area

**Fig. 2:**
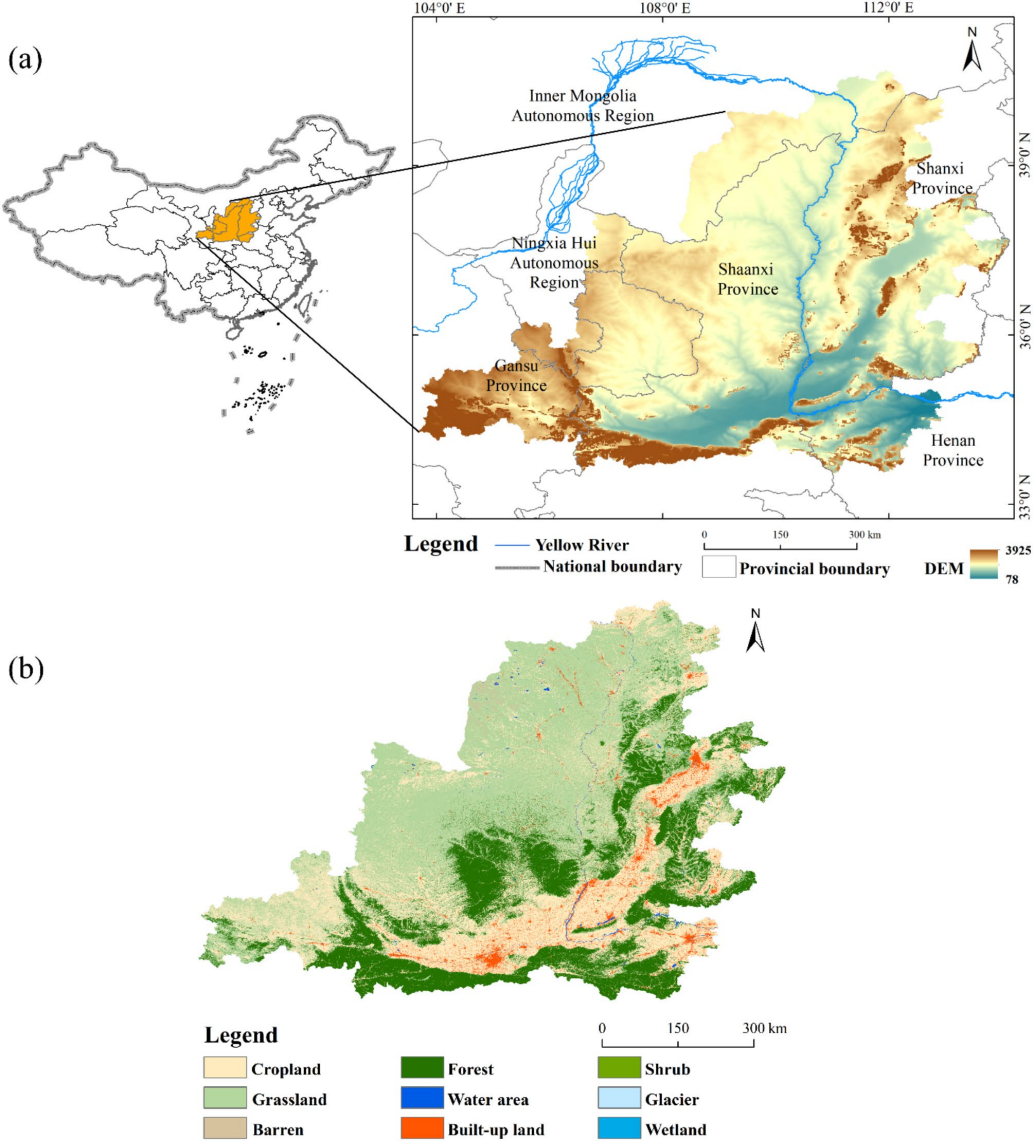
Spatial overview of the study. (**a**) Location in China, and (**b**) Land use type.

The Middle Yellow River Basin extend from Hekou Town in Inner Mongolia in the west to Taohuayu in Zhengzhou, Henan, in the east. The main channel spans 1,206 km, with a basin area of 3.44 × 10^5^km^2^, accounting for 43.27% of the total area of the Yellow River Basin. Based on the natural watershed boundaries defined by the Yellow River Conservancy Commission, combined with studies by Zhao et al.^39^ and Shen et al.^40^, this study area is defined to encompass 226 counties (Fig. 2). The Middle Yellow River Basin represents one of China’s most concentrated areas of population-resource-environment conflicts. This region experiences a continental monsoon climate, with long-term average precipitation ranging from 308.6 to 756.5 mm. Approximately 61% of the area consists of the Loess Plateau, where severe soil erosion has resulted in a fragile ecological environment. Meanwhile, the Middle Yellow River Basin hold the substantial position in the national economic development landscape. This region is not only a major base for energy-intensive industries and a primary agricultural area in China but also rich in historical heritage and tourism resources. Key urban clusters in this area include the Hohhot-Baotou-Ordos-Yulin, central Shanxi, Guanzhong Plain, and Central Plains urban agglomeration. In 2020, the population of the Middle Yellow River Basin was 79.85 million, accounting for 20.24% of the total population in the Yellow River Basin, with the urbanization rate of 49.77%.

### Data sources

This study selects 2010, 2015, and 2020 as time points, using the county-level administrative divisions in the Yellow River Basin as of 2020 as the geographic reference units, resulting in a comprehensive dataset of 226 counties. County-level administrative vector data were obtained from the National Vector Dataset of China provided by the National Geomatics Center of China (https://www.ngcc.cn/ngcc/). Land use data were sourced from the Annual China Land Cover Dataset by Wuhan University (https://zenodo.org/record/5816591), with a resolution of 30 m × 30 m. The Digital Elevation Model (DEM) was obtained from the Geospatial Data Cloud (http://www.gscloud.cn/) at a resolution of 30 m × 30 m. Net Primary Productivity (NPP) data were sourced from the Google Earth Engine data platform (https://lpdaac.usgs.gov/products/mod17a3hgfv061/). PM2.5 data were obtained from the Atmospheric Composition Analysis Group at Dalhousie University in Canada (http://fizz.phys.dal.ca/). Socio-economic data were sourced from the *China County Statistical Yearbook*, *China Science and Technology Statistical Yearbook*, *Compilation of National Agricultural Product Cost–Benefit Data*, as well as statistical yearbooks and bulletins from provincial and municipal statistics bureaus. Any adjustments related to administrative divisions were based on government statistical data for the relevant years.

### Research methods

#### Ecological environment index

Ecosystem service value is the crucial indicator of ecological conditions, and this study uses ecosystem service value to represent the level of ecological environment. The equivalent factor method proposed by Xie et al.^41^ was developed into an ecosystem service value system adapted to China’s specific context. Based on the per-unit area equivalent factor evaluation method and its adjustments, the ecosystem service value for counties in the Middle Yellow River Basin is calculated and standardized to obtain an ecological environment index^42^.

##### Adjustment of equivalent factors

One standard unit of ESV is defined as one-seventh of the annual economic value of the average grain yield per hectare of farmland. Based on the *Compilation of National Agricultural Product Cost–Benefit Data*, the primary grain crops and corresponding prices for each province and year are identified. To mitigate the impact of price fluctuations across different years, the CPI was calculated for correction. The equivalent factors for each year were multiplied by 129%, 113%, and 100%, respectively, to obtain the value of one standard unit of ESV equivalent factor (Table 1). The formula is as follows:

1 $$E_{a} = 1/7\sum\limits_{i = 1}^{n} {\frac{{m_{i} p_{i} q_{i} }}{M}}$$

where ${E}_{a}$ represents the economic value of one unit of ecosystem service in the study area (CNY/hm^2^); *i* is the type of grain crop; ${m}_{i}$ is the average price of the *i*-th grain crop (CNY/kg); ${p}_{i}$ is the yield per unit area of the *i*-th grain crop (kg/hm^2^); ${q}_{i}$ is the sown area of the *i*-th grain crop (hm^2^); and *M* is the total sown area for all grain crops (hm^2^).

**Table 1:** Table of adjustment coefficients for ecosystem service value.

Regions	Adjusted equivalent factor (CNY/hm^2^)			Biotic factor adjustment coefficient		
	2010	2015	2020	2010	2015	2020
Shanxi	1443.3	1382.2	1829.4	0.58	0.63	0.78
Shaanxi	1514.9	1510.6	1802.7	0.70	0.76	0.87
Inner Mongolia	1700.4	1847.4	2272.8	0.46	0.50	0.38
Ningxia	1539.7	1453.7	2079.2	0.47	0.56	0.50
Gansu	1178.1	1238.8	1625.9	0.58	0.63	0.71
Henan	2045.8	2126.4	2159.9	0.72	0.80	0.97

#### Adjustment of unit area ESV coefficients for different land use types

Based on the characteristics of land spatial changes and with reference to the modified unit area service value equivalents of China’s terrestrial ecosystems proposed by Xie et al.^41^, the basic table of ESV per unit area for the Middle Yellow River Basin is established (Table 2). Since variations in vegetation density within the same land use type can lead to differences in ecosystem service levels, this study further refines the ESV by applying the biomass factor adjustment coefficient (Table 1), yielding the ESV for the Middle Yellow River Basin. The formulas are as follows:

2 $$ESV = \sum\limits_{i = 1}^{n} {\sum\limits_{j = 1}^{n} {\sum\limits_{k = 1}^{n} {A_{ij} } } } B_{ik} C_{k}$$

3 $$A_{ij} = E_{a} \times Y_{ij} \times CPI$$

4 $$C_{k} = \frac{{NPP_{k} }}{{\overline{{NPP_{k} }} }}$$

where ${A}_{ij}$ represents the value coefficient of the *j*-th ecosystem service for the *i*-th land use type; ${B}_{ij}$ represents the area of the *i*-th land use type in the *k*-th region; ${C}_{k}$ is the biomass factor adjustment coefficient for the *k*-th region; $\overline{{{\text{NPP}}_{{\text{k}}} }}$ is the national average Net Primary Productivity; ${NPP}_{k}$ is the Net Primary Productivity value for the *k*-th region; *i*, *j*, and *k* represent the land use type, ecosystem service type, and research unit, respectively; ${E}_{a}$ is the economic value of one unit of ecosystem service in the study area (CNY/hm^2^); ${Y}_{ij}$ is the equivalent value of the *j*-th ecosystem service for the *i*-th land use type.

**Table 2:** Unit area ecosystem service value equivalent.

Ecosystem service functions		Land use types							
Type 1	Type 2	Cropland	Forest	Shrub	Grassland	Water	Glacier	Barren	Wetland
Provision services	Food products	0.85	0.27	0.19	0.23	0.80	0	0.005	0.51
	Material products	0.40	0.63	0.43	0.34	0.23	0	0.015	0.50
	Water supply	0.02	0.33	0.22	0.19	8.29	2.16	0.010	2.59
Regulating services	Gas regulation	0.67	2.07	1.41	1.21	0.77	0.18	0.065	1.90
	Climate regulation	0.36	6.20	4.23	3.19	2.29	0.54	0.050	3.60
	Environmental purification	0.10	1.80	1.28	1.05	5.55	0.16	0.705	3.60
	Hydrological regulation	0.27	3.86	3.35	2.34	102.24	7.13	0.120	24.23
Supporting services	Soil conservation	1.03	2.52	1.72	1.47	0.93	0	0.075	2.31
	Maintenance of nutrient cycling	0.12	0.19	0.13	0.11	0.07	0	0.005	0.18
	Maintenance of biodiversity	0.13	2.30	1.57	1.34	2.55	0.01	0.070	7.87
Cultural services	Aesthetic landscape	0.06	1.01	0.69	0.59	1.89	0.09	0.030	4.73

#### Evaluation indicator system for high-quality economic development

Based on the actual conditions of counties in the Middle Yellow River Basin and referring to relevant studies^43–45^, the evaluation indicator system for high-quality economic development is constructed, which includes five dimensions: innovation, coordination, green, openness, and shared development (Table 3). Innovation Development reflects the sustained driving force for high-quality economic growth, measured by indicators such as the number of patents per ten thousand people, the number of enterprises above designated scale, and the industrial structure (ratio of the secondary to tertiary industries). Coordination Development emphasizes the overall efficiency of growth, represented by per capita GDP, fiscal self-sufficiency rate, and urban–rural disparity (the ratio of urban to rural disposable income). Green Development is the fundamental requirement for high-quality economic development, assessed by PM2.5 concentration, forest coverage rate, and agricultural fertilizer application intensity. Openness Development reflects the new development pattern of domestic and international dual circulation, primarily represented by market activity (the proportion of retail sales of consumer goods to GDP) and per capita import and export volume. Shared Development seeks to achieve inclusive economic and social benefits, with key indicators including the number of students in compulsory education, the number of hospital and health center beds, and the number of beds in social welfare institutions.

**Table 3:** Evaluation indicator system for high-quality economic development.

Primary index	Secondary index	Weight/%	Attribute
Innovation development	number of patents per ten thousand people(X1)	14.3	+
	number of enterprises above designated scale(X2)	8.2	+
	industrial structure(X3)	8.9	+
Coordination development	per capita GDP(X4)	6.9	+
	urban–rural disparity(X5)	1.2	−
	fiscal self-sufficiency rate(X6)	5.9	+
Green development	PM2.5 concentration(X7)	1.2	−
	agricultural fertilizer application intensity(X8)	1.1	−
	forest coverage rate(X9)	8.7	+
Openness development	market activity(X10)	3.5	+
	per capita import and export volume(X11)	17.6	+
Shared development	number of students in compulsory education(X12)	4.5	+
	number of hospital and health center beds(X13)	7.5	+
	number of beds in social welfare institutions(X14)	10.5	+

" + " denotes the positive attribute of the indicator, while " − " represents the negative attribute of the indicator.

The entropy method objectively reflects the utility value of the information entropy of indicators, thereby effectively reducing the interference of subjective factors. This method calculates entropy weights primarily based on the degree of variation and information entropy of each indicator, resulting in more objective indicator weights. This study employs the entropy method and the linear weighting method to conduct a comprehensive assessment of the level of high-quality economic development.

#### Coupling coordination degree model

The Coupling Coordination Degree Model can quantitatively evaluate the degree of interaction and coordination between the ecological environment and high-quality economic development. By using the main functional zoning as the basis, the importance of ecological environment and high-quality economic development is classified, allowing for a more accurate measurement of the coupling coordination level between the two. The formulas are as follows:

5 $$C = \frac{{2\sqrt {EE \times HQED} }}{EE + HQED}$$

6 $$T = \alpha EE + \beta HQED$$

7 $$D = \sqrt {C \times T}$$

where *C* represents the coupling degree, reflecting the interaction between the ecological environment and high-quality economic development; *EE* is the Ecological Environment Index; *HQED* is the High-Quality Economic Development Index; *T* is the coordination degree index, reflecting the overall development level of both systems; *α* and *β* represent the relative importance of ecological environment and high-quality economic development, determined based on the main functional zoning and existing research foundation^46^; *D* is the coupling coordination degree, which provides a comprehensive evaluation of the development status of the two systems, and classifies it into four categories (Table 4).

**Table 4:** Types of coupling coordination degree.

Type	Coupling coordination degree	Relative value	Lag type
Seriously unbalanced	0 ≤ D < 0.32	ESV—HQED > 0.1	Antagonistic (High-Quality Economic Development Lagging)
		ESV—HQED < -0.1	Antagonistic (Ecological Environment Lagging)
		-0.1 ≤ ESV—HQED ≤ 0.1	Low-Level harmony
Slightly unbalanced	0.32 ≤ D < 0.41	ESV—HQED > 0.1	Antagonistic (High-Quality Economic Development Lagging)
		ESV—HQED < -0.1	Antagonistic (Ecological Environment Lagging)
		-0.1 ≤ ESV—HQED ≤ 0.1	Low-Level harmony
Slightly balanced	0.41 ≤ D < 0.50	ESV—HQED > 0.1	Antagonistic (High-Quality Economic Development Lagging)
		ESV—HQED < -0.1	Antagonistic (Ecological Environment Lagging)
		-0.1 ≤ ESV—HQED ≤ 0.1	High-Level harmony
Moderately balanced	0.50 ≤ D < 1	ESV—HQED > 0.1	Antagonistic (High-Quality Economic Development Lagging)
		ESV—HQED < -0.1	Antagonistic (Ecological Environment Lagging)
		-0.1 ≤ ESV—HQED ≤ 0.1	High-Level harmony

#### Exploratory spatial data analysis

Exploratory Spatial Data Analysis includes both global spatial autocorrelation and local spatial autocorrelation. Global spatial autocorrelation is used to reflect the distribution characteristics of geographic observations across the entire spatial domain, while local spatial autocorrelation reflects the spatial variation and significance between each area and its neighboring regions. In this study, the spatial distribution pattern of the coupling coordination degree is analyzed through global and local spatial autocorrelation models. The formulas are as follows:

8 $$I = \frac{{n\sum\limits_{i = 1}^{n} {\sum\limits_{j = 1}^{m} {W_{ij} } } (X_{i} - X^{\prime } )(X_{j} - X^{\prime } )}}{{\sum\limits_{i = 1}^{n} {\sum\limits_{j = 1}^{m} {W_{ij} } } \sum\limits_{i = 1}^{n} {(X_{i} - X^{\prime } )^{2} } }}$$

9 $$I^{\prime} = \frac{{n(X_{i} - X^{\prime } )\sum\limits_{j = 1}^{m} {W_{ij} } (X_{j} - X^{\prime } )}}{{\sum\limits_{i = 1}^{n} {\left( {X_{i} - X^{\prime } } \right)} }}$$

where *I* is the global autocorrelation index, ranging from [-1,1]. $I^{\prime }$ represents the local autocorrelation index; ${X}_{i}$ and ${X}_{j}$ are the indicator values for attribute units *i* and *j*, respectively; ${W}_{ij}$ is the spatial weight matrix.

#### Random forest (RF) model

The healthy ecological environment relies on the driving effect of high-quality economic development. Before assessing the driving mechanism of high-quality economic development on the ecological environment, it is essential to address any multicollinearity among the indicators. This can be evaluated using the Variance Inflation Factor (VIF) for each indicator. The results indicate that the VIF values of all indicators are less than 5. The formula is as follows:

10 $$VIF = \frac{1}{{1 - R_{i}^{2} }}$$

where $VIF$ is the Variance Inflation Factor, ${R}_{i}$ represents the correlation coefficient of the regression analysis of the independent variable ${X}_{i}$ on other variables. The higher $VIF$ indicates greater multicollinearity among the independent variables.

The RF model is employed to assess the importance of independent variables. RF model is a non-parametric ensemble learning model that consists of numerous decision trees, offering advantages such as rapid processing, high model performance, ease of parameter optimization, and simplicity of implementation^47^. The contribution level in the RF model is calculated independently for each feature based on the percentage increase in mean squared error. As a result, the total contribution does not necessarily sum to 100% because multiple features can act simultaneously during node splitting, rather than each feature acting in isolation. When interpreting this model, the primary focus is on the contribution level of each feature. The formulas are as follows:

11 $$VIM_{j}^{(OOB)} = \frac{{\sum\limits_{P = 1}^{{n_{O}^{i} }} I (Y_{P} = Y_{P}^{i} )}}{{n_{o}^{i} }} - \frac{{\sum\limits_{P = 1}^{{n_{O}^{i} }} I (Y_{P} = Y_{{P,\pi_{j} }}^{i} )}}{{n_{o}^{k} }}$$

12 $$VIM_{j}^{(OOB)} = \frac{{\sum\limits_{i = 1}^{n} V IM_{ij}^{(OOB)} }}{n}$$

where ${n}_{o}^{i}$ represents the number of out-of-bag (OOB) observations for the *i*-th tree. *I*(*g*) is an indicator function that takes a value of 1 when two values are equal and 0 when they are not. ${Y}_{P}\in \left\{0,\left.1\right\}\right.$ represents the actual result of the *p*-th observation. ${Y}_{P}^{i}\in \left\{0,\left.1\right\}\right.$ represents the prediction for the *p*-th observation of the OOB data by the *i*-th tree before random replacement. ${Y}_{P,\pi j}^{i}\in \left\{0\right.,\left.1\right\}$ denotes the prediction result for the *p*-th observation of the OOB data by the *i*-th tree after random replacement. *n* represents the number of classification trees.

#### Geographical and temporal weighted regression (GTWR) model

Due to the spatial heterogeneity of the driving mechanism of high-quality economic development on the ecological environment, statistical analysis models may overlook the spatial distribution characteristics of driving factors. Therefore, the Ordinary Least Squares (OLS), Geographically Weighted Regression (GWR), and Geographical and Temporal Weighted Regression (GTWR) model are selected for simulation and evaluation (Table 5). The coefficient of determination *R*^2^ of the GTWR model reaches 0.913, which is higher than that of the other models; the AICc is -870.07, significantly lower than that of the other models. The GTWR model is employed to explore the effects of various driving factors on the ecological environment.

**Table 5:** Relevant parameters of models.

Models	*R*^2^	Adjusted *R*^2^	AICc
OLS	0.685	0.673	− 257.78
GWR	0.773	0.766	− 693.41
GTWR	0.913	0.902	− 870.07

The GTWR model builds upon the GWR model by incorporating the time dimension. This model captures variations in parameters from both temporal and spatial dimensions, allowing for the identification of spatiotemporal non-stationarity. The formula is as follows:

13 $$Y_{i} = \beta_{0} (u_{i} ,v_{i} ,t_{i} ) + \sum\limits_{k = 1}^{n} {\beta_{k} } (u_{i} ,v_{i} ,t_{i} )X_{ik} + \varepsilon_{i}$$

where ${Y}_{i}$ is the dependent variable; ${X}_{i}$ is the independent variable; *i* represents the sample region; *u*, *v* are the coordinates of the sample region; *t* is the time; ${\beta }_{0}({u}_{i},{v}_{i},{t}_{i})$ is the intercept term; ${\beta }_{k}({u}_{i},{v}_{i},{t}_{i})$ is the estimated coefficient of the dependent variable; ${\varepsilon }_{i}$ is the random error term; *n* is the number of explanatory variables.

## Results

### Comprehensive analysis of EE and HQED level

#### Spatiotemporal changes in the ecological environment

**Fig. 3:**
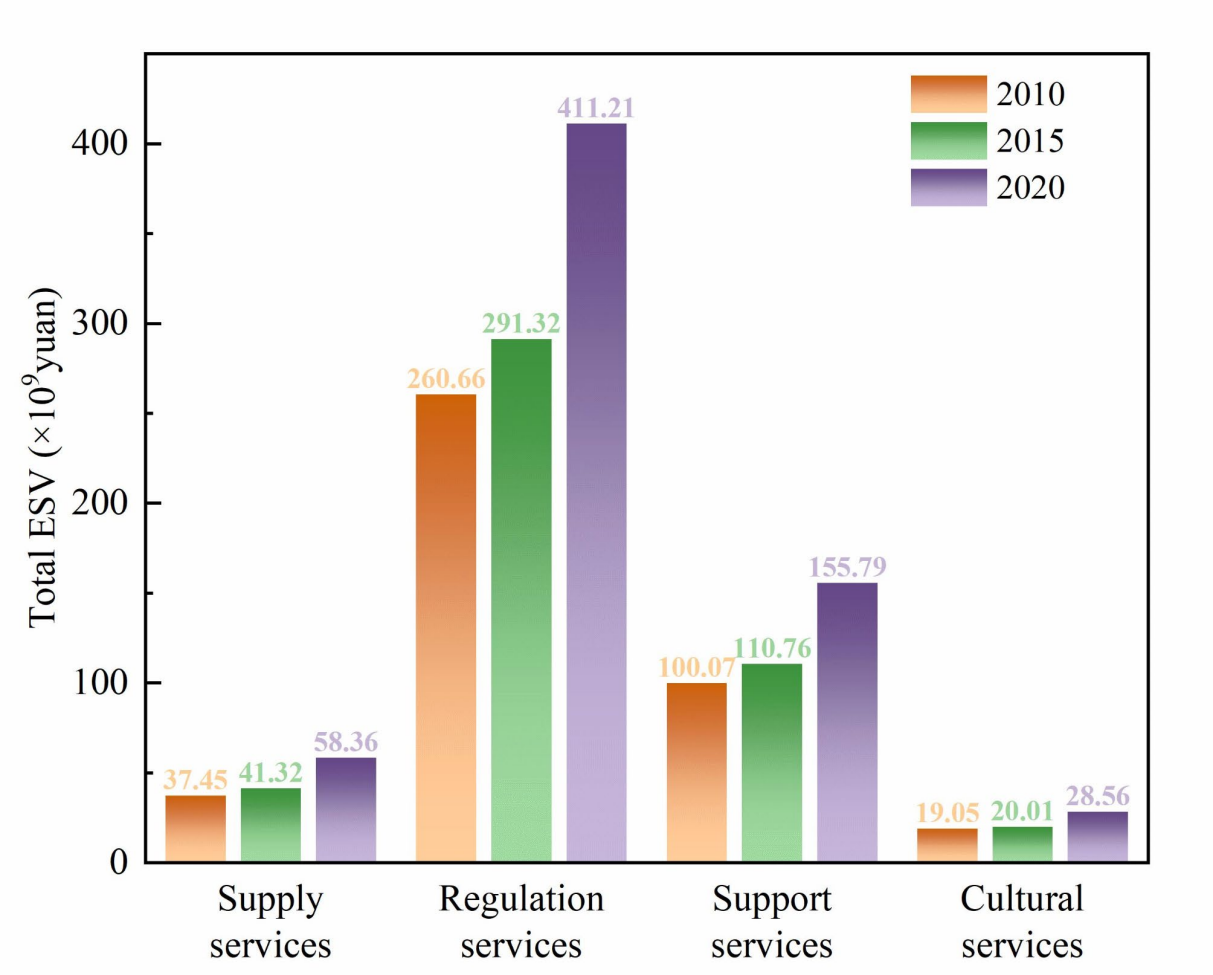
The value of different service functions of ecosystems in the Middle Yellow River Basin.

During the study period, the average ecological environment index for the counties in the Middle Yellow River Basin were 0.22, 0.21, and 0.23, respectively, indicating an overall stable and improving ecological environment. From the temporal perspective, the period from 2010 to 2015 was characterized by ecological degradation, with a decrease of 4%, reflecting a slight decline in regional ecosystem service levels. The period from 2015 to 2020, however, marked an ecological optimization phase, with an increase of 9%. Regarding major land use types (Table 6), cropland, forest, and grassland were the main contributors to the ecosystem service value in the Middle Yellow River Basin, with forests providing 46% of the ecosystem service value in 2020. The ecosystem service value of water exhibited the highest increase from 2010 to 2020, rising by 76%. Furthermore, as shown in Fig. 3, supply, regulation, support, and cultural services all followed an upward trend. Among these, regulation services contributed the most to ecosystem service value (411.21 × 10⁹ yuan in 2020), while cultural services contributed the least (28.56 × 10^9^ yuan in 2020).

**Table 6:** Changes in ESV for major land use types.

Types	ESV (10^9^ yuan)			Growth rate (%)		
	2010	2015	2020	2010–2015	2015–2020	2010–2020
Cropland	48.08	50.58	72.41	5.20	43.16	50.60
Forest	178.97	202.22	303.25	12.99	49.96	69.44
Shrub	1.69	1.62	1.76	-4.14	8.64	4.14
Grassland	171.38	189.33	246.78	10.47	30.34	43.99
Water	16.77	19.43	29.52	15.86	51.93	76.02
Barren	0.32	0.23	0.21	-28.12	-8.69	-34.37

**Fig. 4:**
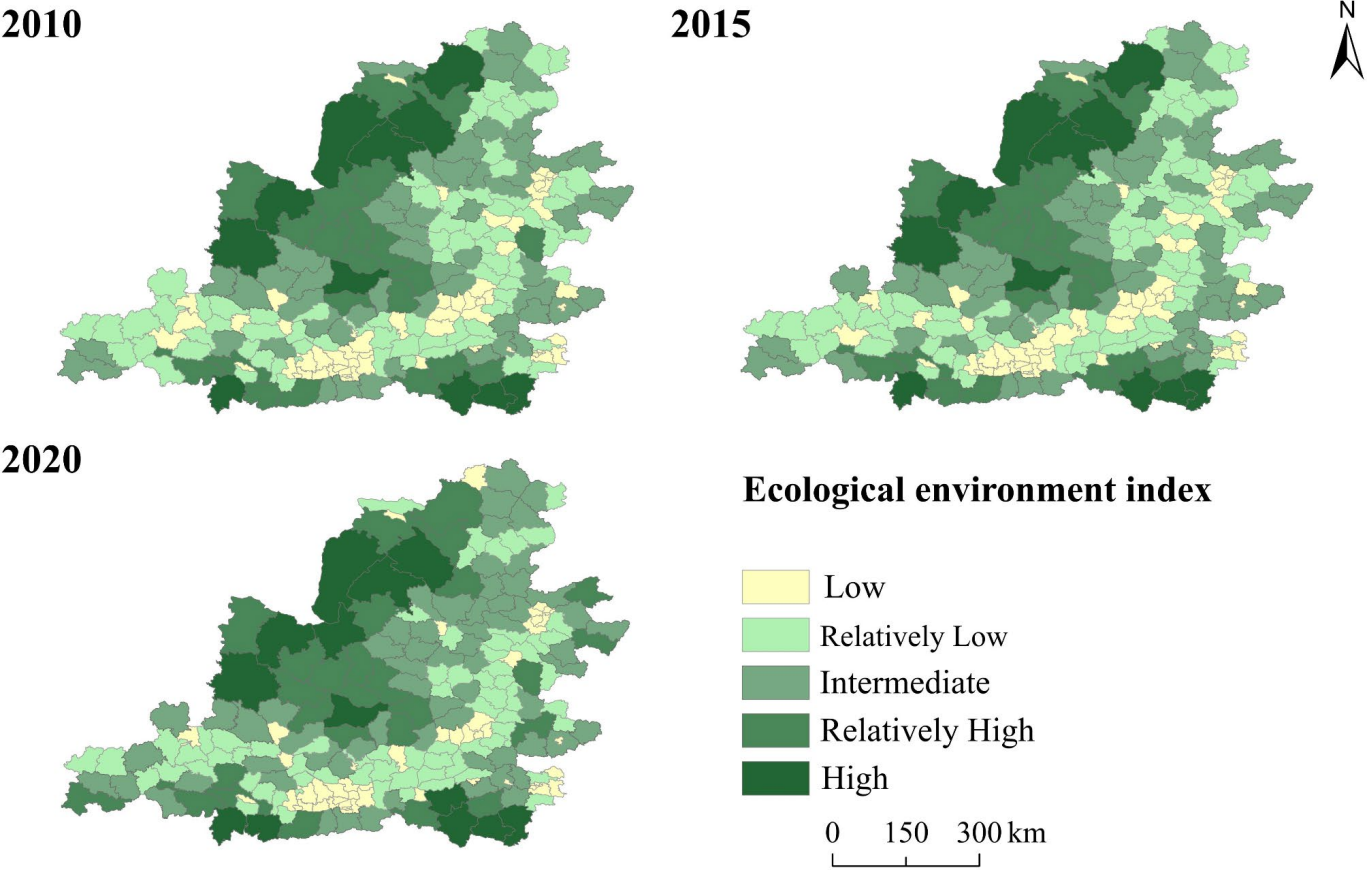
Spatiotemporal differentiation characteristics of EE in the Middle Yellow River Basin.

From the spatial perspective (Fig. 4), counties with low level of ecological environment are mainly distributed in the central and southwestern parts of Shanxi and the central region of Shaanxi, and have gradually decreased over time. Among these, Taiyuan and Xi’an are the main low-level concentration areas. As central cities, the large population agglomeration and rapid expansion of built-up areas have impacted the ecological quality. The areas with high and relatively high levels of ecological environment are mainly located in the northwest counties, gradually expanding eastward over time. It is worth noting that ecological environment of counties such as Yuyang District, Dingbian County, and Fu County has consistently remained at high level, indicating their superior ecological foundations.

#### Spatiotemporal changes in the high-quality economic development

**Fig. 5:**
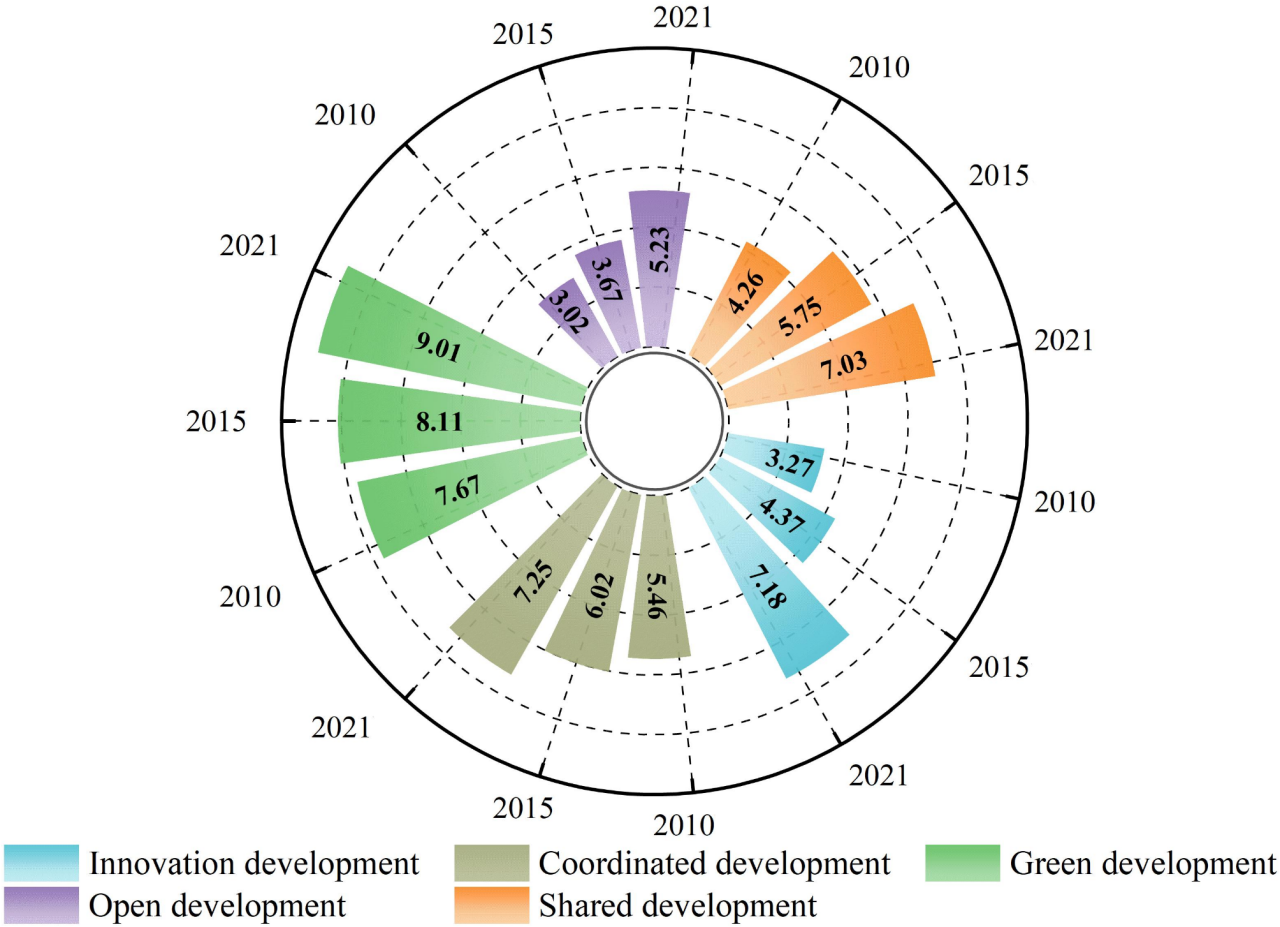
Temporal variations in different aspects of HQED in the Middle Yellow River Basin.

The average high-quality economic development index for the counties in the Middle Yellow River Basin during the study period was 0.11, 0.12, and 0.16, showing a gradually increasing trend. From the temporal perspective, the period from 2010 to 2015 was the phase of slow growth, with a growth rate of 9%. The period from 2015 to 2020 was the phase of rapid growth, with a growth rate of 33%. As shown in Fig. 5, the five dimensions of innovation, coordination, green, openness, and shared development all show the continuous upward trend. Among them, green development reached its highest value during the study period, indicating that the national policy of resource conservation and environmental protection has been increasingly implemented. From 2010 to 2015, shared development had the highest growth, with an increase of 35%; from 2015 to 2020, innovation development saw the highest growth, with an increase of 64%.

**Fig. 6:**
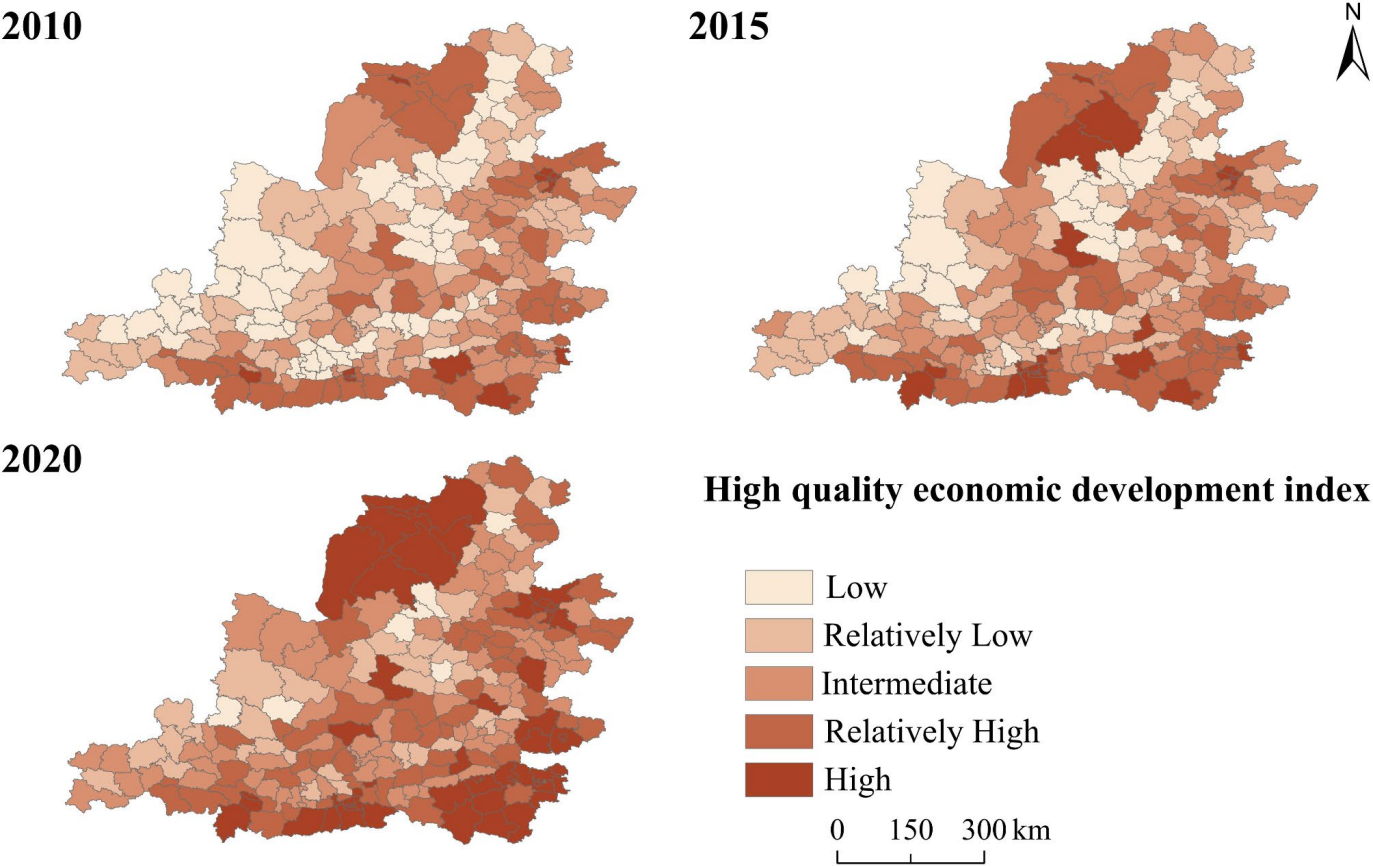
Spatiotemporal differentiation characteristics of HQED in the Middle Yellow River Basin.

From the spatial perspective, the overall distribution shows pattern of higher values in the southeast and lower values in the northwest (Fig. 6). In 2010, the high-quality economic development level of the Middle Yellow River Basin presented the spatial pattern of higher in the southeast, lower in the central and western regions. Among them, 57 counties were categorized as low-level, accounting for 25.2% of the total. In 2015, the number of counties with low-level development decreased, mainly distributed in the Shanxi-Shaanxi boundary area, as well as some counties in Gansu. In contrast, the high-level counties expanded gradually from the southeast to the northwest. In 2020, the number of low-level counties further decreased to 6, while the number of high-level counties increased to 61, accounting for 26.9% of the total. High-level counties were predominantly concentrated in regional center cities. The radiating effect of urban agglomerations considerably intensified, and the regional disparities gradually decreased.

### Spatiotemporal differentiation characteristics of coupling relationship

#### Temporal changes in the coupling coordination degree

The coupling coordination degree between EE and HQED shows an overall upward trend. In 2010, the number of counties in the slightly balanced and moderately balanced stages was 55, accounting for 24% of the total counties in the Middle Yellow River Basin, while 171 counties were in the seriously unbalanced and slightly unbalanced stages. By 2015, the number of counties in the slightly balanced and moderately balanced stages was 54, while the number of counties in the seriously unbalanced and slightly unbalanced stages reached 172. In 2020, the number of counties in the slightly balanced and moderately balanced stages increased to 89, accounting for 39% of the total, while the number of counties in the seriously unbalanced and slightly unbalanced stages decreased to 137. This indicates that the period from 2010 to 2015 was stable phase for coupling coordination, with no considerable changes between the unbalanced and balanced counties. From 2015 to 2020, however, the coupling coordination degree rose, with the number of balanced counties increasing by 35, reflecting the 65% growth.

#### Spatial variation of coupling coordination degree

**Fig. 7:**
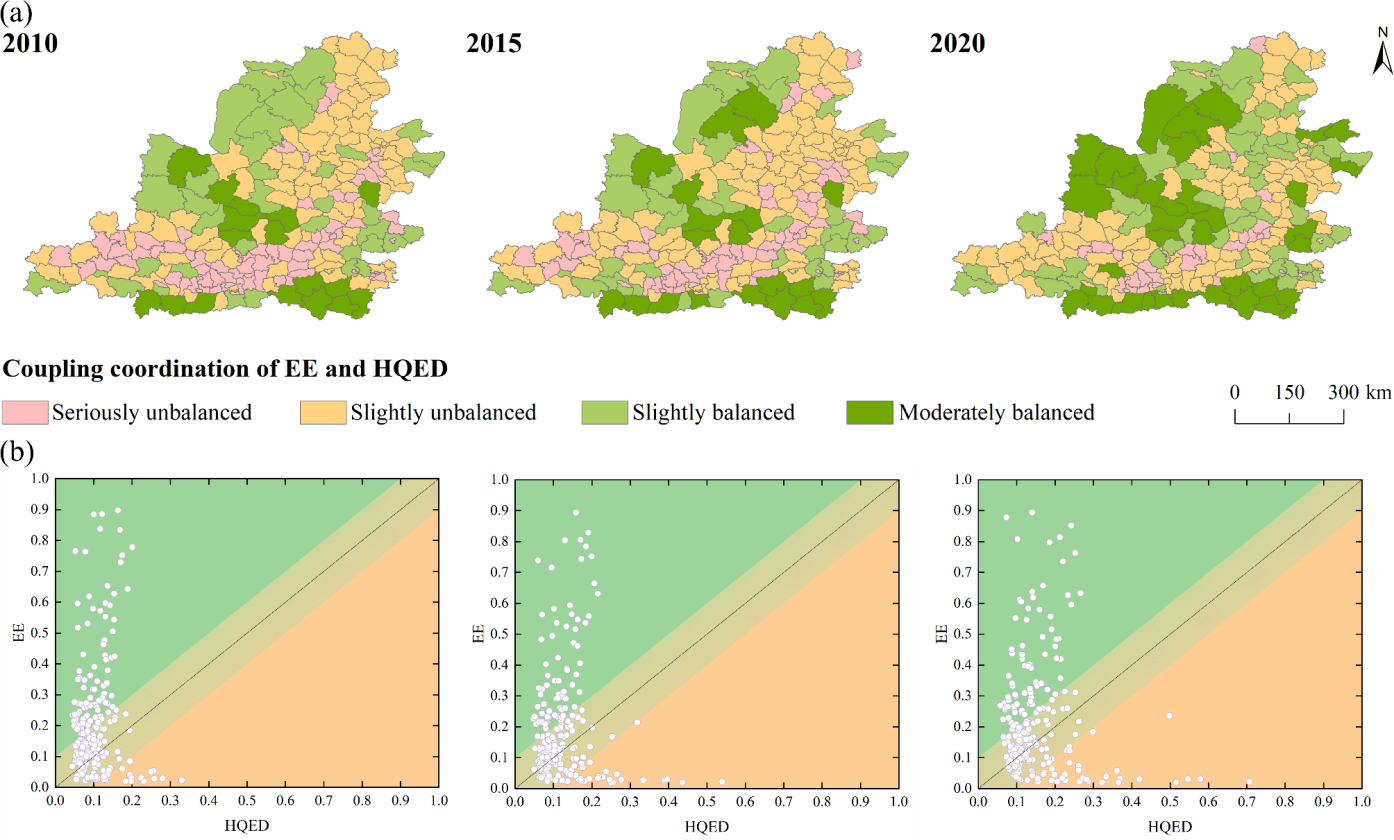
(**a**) Spatiotemporal distribution of coupling coordination types between EE and HQED, and (**b**) Relative development degree.

The coupling coordination degree of counties in the Middle Yellow River Basin shows a spatial distribution pattern of high in the southeast and northwest, low in the central region (Fig. 7a). Among them, the seriously imbalanced counties are mostly concentrated in Shanxi and Shaanxi provinces. In the southwestern part of Shanxi Province, counties are predominantly characterized by resource-dependent industries, which are associated with prominent issues of environmental pollution and ecological degradation. In the central part of Shaanxi Province, with the rapid expansion of construction land, constraints on resources and the environment still persist. In 2010, the balanced centers were mainly concentrated in counties such as Luanchuan and Taibai, while the seriously unbalanced areas were concentrated in the central part of Shaanxi. By 2015, the overall balanced level had not changed considerably compared to 2010, but the number of seriously unbalanced counties in the central region decreased and shifted to the slightly unbalanced stage. In 2020, the range of balanced areas expanded, but most counties still belonged to the slightly unbalanced areas. Overall, the unbalanced counties became increasingly dispersed in their distribution, while the balanced counties formed agglomerated distributions. Unbalanced counties were often those where both ecological environment and high-quality economic development were lagging, while balanced counties were mostly those with delayed economic development. This is because many counties are located in areas suffering from soil erosion or are agricultural zones with lagging economic development. The ecological lagging is predominantly concentrated in provincial capitals and urban districts.

Analyzing from the perspective of relative development level provides a clearer comparison between EE and HQED (Fig. 7b). Most counties are concentrated in the low-level harmony and high-level harmony categories, where the difference between the EE index and the HQED index lies within the range of -0.1 to 0.1. From 2010 to 2020, the number of low-level harmony counties decreased from 110 to 86, while the number of high-level harmony counties increased from 10 to 22. At the same time, in the antagonistic-type counties, there are more counties with lagging HQED than those with lagging EE. From 2010 to 2020, the number of counties with lagging HQED decreased from 91 to 84, while the number of counties with lagging EE increased from 15 to 34. Most counties in mountainous areas are above the standard line, indicating that the EE is leading. Over time, some economically developed regions have gradually seen their HQED outpace the EE.

### Spatial correlation characteristics of coupling coordination degree

**Fig. 8:**
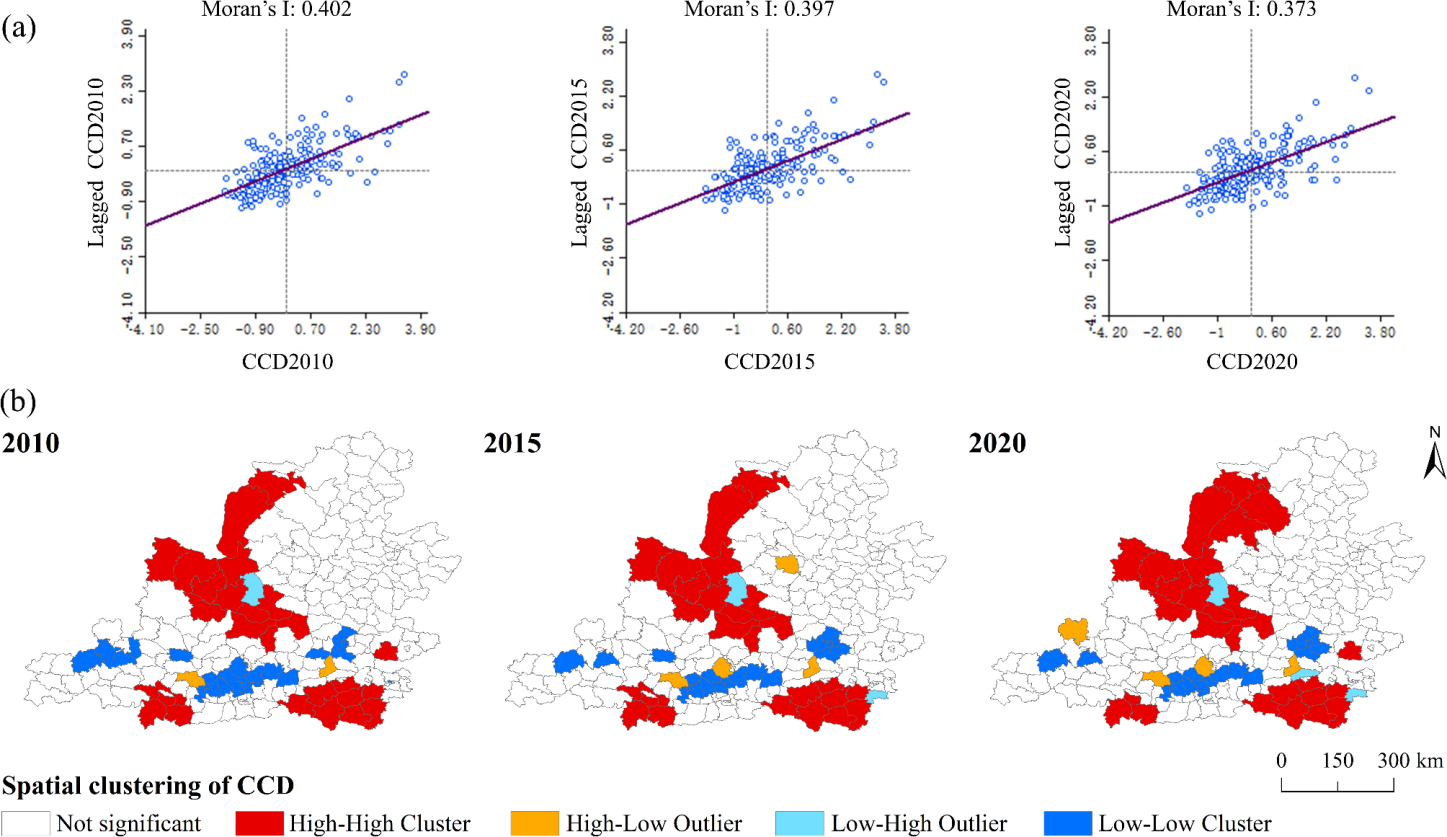
(**a**) Coupling coordination degree Moran’s I scatter plot. (**b**) Spatiotemporal change in the LISA cluster of CCD between EE and HQED. ("High-High Cluster": aggregation zones with high coupling coordination levels; "High-Low Outlier": high coordination areas adjacent to low coordination clusters; "Low-Low Cluster": agglomerations exhibiting low coupling coordination intensities; "Low–High Outlier": low coordination regions neighboring high coordination areas).

Using GeoDa software, the spatial autocorrelation of the coupling coordination degree between EE and HQED was calculated. The Global Moran’s I index for 2010–2020 were 0.402, 0.397, and 0.373, respectively, all greater than 0; the z-values were 9.595, 9.698, and 9.034, all greater than 1.96; and the p-values were all less than 0.01. Significance tests indicate that there is a significant spatial correlation in the coupling coordination degree (Fig. 8a). The overall trend of the Global Moran’s I value shows downward slope, suggesting that the spatial agglomeration effect has weakened and the spatial distribution differences are gradually becoming more balanced. The counties primarily cluster in the first and third quadrants, with spatial distribution patterns reflecting "High-High" and "Low-Low" agglomeration. The number of counties in the second and fourth quadrants is relatively small, indicating strong positive spatial correlation between EE and HQED coupling coordination degree.

Using the Local Spatial Autocorrelation (LISA) model, the local spatial correlation tables of the coupling coordination degree between EE and HQED for 2010, 2015, and 2020 were obtained (Table 7) along with the LISA cluster maps (Fig. 8b). The results were categorized into five types: High-High Cluster, High-Low Outlier, Low–High Outlier, Low-Low Cluster, and not significant areas. Throughout the study period, there were no considerable spatial jumps in the local LISA spatial agglomeration areas. The number of counties in High-High agglomeration areas decreased from 26 to 25, then increased to 27. These are mainly distributed in regions such as Wuqi County in the northwest and Luoning County in the south. These areas exhibit a high level of coordination between the EE and HQED, forming a high-coordination radiation agglomerate. Over time, the spatial scope of these areas has gradually expanded, indicating that the coupling coordination level in surrounding counties is continuously improving. The number of counties in Low-Low agglomeration areas decreased from 25 to 20, with the gradually shrinking spatial range. These are primarily located in regions such as Wenxi County in the south-central area and Tongwei County in the west. In these areas, both ecological environment and high-quality development levels are lagging, forming low-coordination contraction. The number of counties in High-Low agglomeration areas increased from 2 to 4, suggesting the emergence of potential points of radiation. The number of counties in Low–High agglomeration areas increased from 1 to 3, with Ansai District remaining unchanged throughout the study period, characterized by low coupling coordination level surrounded by counties with high coupling coordination levels.

**Table 7:** Table of local spatial association for coupling coordination degree.

Types	2010		2015		2020	
	Number	Proportion (%)	Number	Proportion (%)	Number	Proportion (%)
H–H agglomeration	26	11.5	25	11.1	27	11.9
H–L agglomeration	2	0.9	4	1.8	4	1.8
L–H agglomeration	1	0.4	2	0.9	3	1.3
L–L agglomeration	25	11.1	21	9.3	20	8.8
Not significant	172	76.1	174	77	172	76.1

### Driving mechanism analysis

#### Contribution degree detection of driving factors

**Fig. 9:**
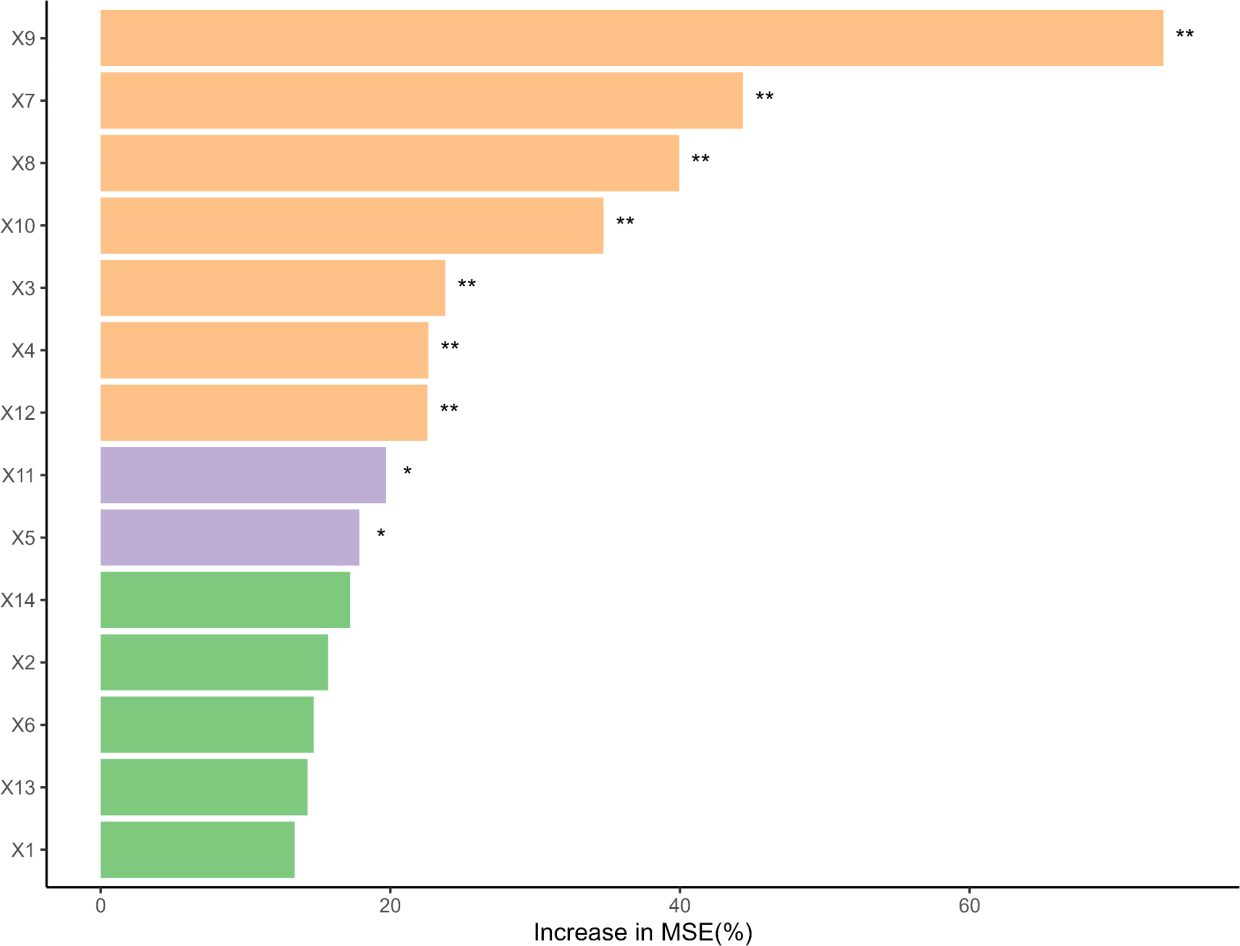
Contribution of driving factors. (The symbol "**" indicates a significance level of *P* < 0.01. The symbol "*" indicates a significance level of *P* < 0.05.)

To detect multicollinearity among the indicators of high-quality economic development, the VIF was applied, revealing that all indicators had VIF values less than 5 (Table 8). After eliminating multicollinearity, the RF model was used to assess the contribution of various factors of HQED to the EE (Fig. 9). The results show that forest coverage rate, PM2.5 concentration, agricultural fertilizer application intensity, market activity, industrial structure, per capita GDP, and number of students in compulsory education all have significant impacts on the ecological environment (*P* < 0.01). Notably, forest coverage rate, PM2.5 concentration, agricultural fertilizer application intensity, and market activity each contribute more than 30%, making them key drivers of the EE. Additionally, per capita import and export volume, and the urban–rural disparity also show significant effects on the EE (*P* < 0.05). The other variables did not have significant effects on the EE (*P* > 0.05). Consequently, only the factors with significant impacts on the EE (*P* < 0.05) were selected for spatiotemporal heterogeneity analysis.

**Table 8:** Variance inflation factor.

Factors	VIF	Factors	VIF	Factors	VIF	Factors	VIF	Factors	VIF	Factors	VIF	Factors	VIF
X1	2.362	X2	1.434	X3	1.267	X4	2.310	X5	2.037	X6	2.039	X7	4.928
X8	3.875	X9	1.398	X10	2.167	X11	2.363	X12	1.556	X13	2.340	X14	1.291

#### Spatiotemporal heterogeneity of driving factors

##### Temporal dimension

**Fig. 10:**
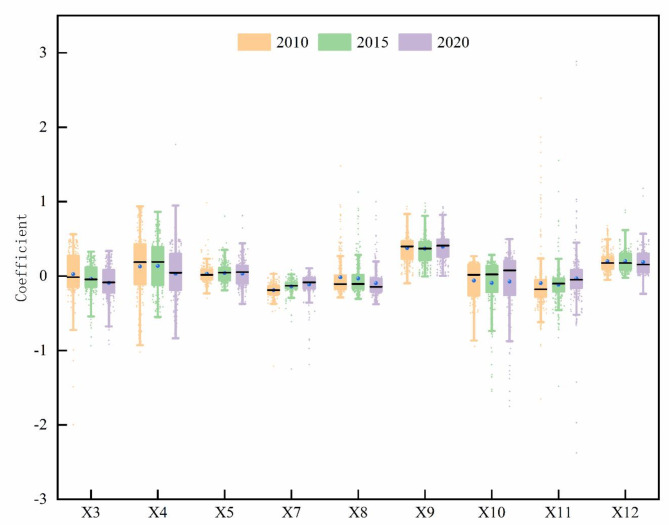
Temporal variations in the regression coefficients of driving factors.

The regression coefficients were calculated using the GTWR analysis module in ArcGIS 10.8, with the bandwidth set to automatic optimization and the spatial distance parameter ratio set to 1. Box plots were generated based on the regression coefficients of different driving factors (Fig. 10).

The factors primarily driving the positive relationship are: forest coverage rate, number of students in compulsory education, per capita GDP, and urban–rural disparity. From 2010 to 2020, the median of the forest coverage rate’s regression coefficient shows a trend of first decreasing and then increasing, dropping from 0.39 in 2010 to 0.36 in 2015, before rising again to 0.40 in 2020. The interquartile range (IQR) fluctuated around 0.24, indicating that the forest coverage rate has the promoting effect on the EE across all counties. The median regression coefficient for the number of students in compulsory education fluctuated between 0.17 and 0.15. The IQR value steadily increased from 0.16 in 2010 to 0.26 in 2020, indicating a gradual increase in the dispersion of regression coefficients. The regression coefficient for per capita GDP generally showed a trend of rising first and then declining, with most values being positive. The IQR fluctuated around 0.5, suggesting considerable differences in the impact of per capita GDP on the EE of different counties. The median value of the regression coefficients for the urban–rural disparity in the Middle Yellow River Basin showed a slight increase near 0, with a relatively low degree of dispersion.

The main negative driving factors for the regression coefficients are: industrial structure, agricultural fertilizer application intensity, per capita import and export volume, PM2.5 concentration, and market activity. From 2010 to 2020, the median regression coefficient of industrial structure for the EE remained relatively stable, with the IQR fluctuating. It decreased from 0.42 in 2010 to 0.31 in 2020. The median regression coefficient of agricultural fertilizer application intensity showed a gradual decline, with a small degree of dispersion. The median regression coefficient of per capita import and export volume showed an increasing trend, from -0.17 in 2010 to -0.05 in 2020. This indicates that the inhibitory effect of per capita import and export volume on the EE has weakened, as most regions’ import and export trade gradually aligns with the concept of green development. The median regression coefficient for PM2.5 concentration fluctuated around -0.13, but its dispersion gradually increased, showing an enhanced inhibitory effect on the EE. The median regression coefficient for market activity rose from 0.02 in 2010 to 0.07 in 2020, with the IQR fluctuating around 0.4, indicating large differences in its impact on the EE across different counties.

##### Spatial dimension

**Fig. 11:**
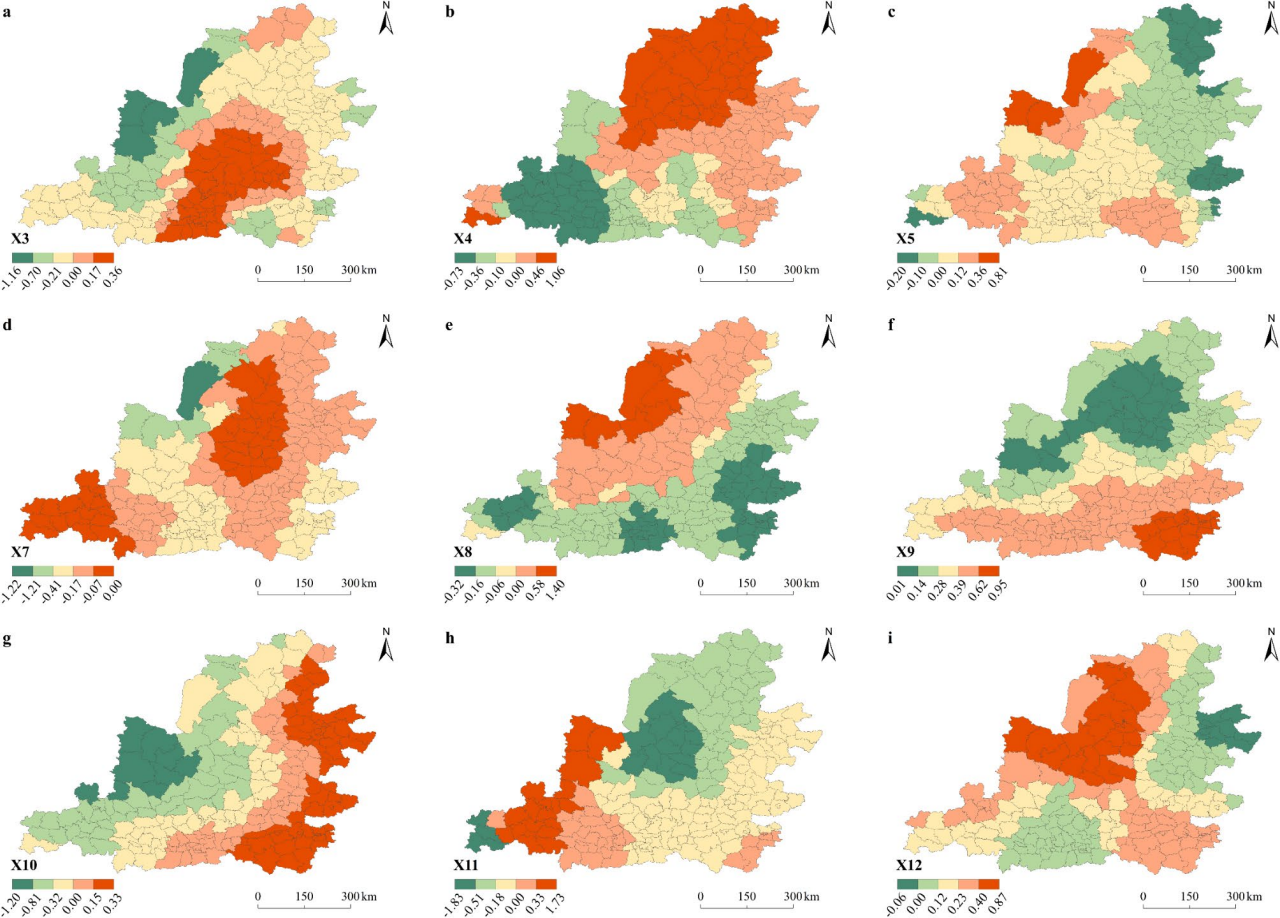
Spatial differentiation characteristics of driving factors.

Utilizing spatial analysis tools to visualize the regression coefficients of driving factors and to analyze the spatial heterogeneity of these factors (Fig. 11).

The impact of industrial structure (X^3^) on the EE showed a circular distribution, gradually weakening from the Guanzhong Plain outward. Areas with strong positive impacts are primarily located in the central and eastern parts of Shaanxi, the southern part of Shanxi, and the southern region of Inner Mongolia. These regions have achieved coordinated development of industrial structure and ecological environment by actively fostering new economic growth areas, such as the cultural tourism industry and clean energy, through industrial transformation and upgrading. Regions with stronger negative impacts are mainly found in the western areas, including parts of Ningxia and Gansu.

The regression coefficients for per capita GDP (X4) are generally distributed in clear pattern, with north–south differences. Positive impact regions are mainly located in most areas of Shanxi, northern Shaanxi, and southern Inner Mongolia. Additionally, small areas in northern Henan, and southern Gansu also show positive impacts. These regions leverage their resource advantages to pursue the eco-economic development path, where economic growth promotes ecological improvements. The negative effects are mainly concentrated in central Shaanxi, eastern Gansu, and southwestern Shanxi.

The impact of urban–rural disparity (X5) on the EE shows east–west differences, with overall trend of increasing impact from east to west. Positive effects are observed in the northwest of Henan, eastern Gansu, and the Ningxia-Shaanxi-Mongolia inter-provincial boundary areas. This indicates that with the reduction of the urban–rural income gap, rural environments have improved, which continues to drive ecological restoration. Negative effects are primarily concentrated in most parts of Shanxi. In these areas, the large urban–rural disparity persists, and the ecological environment remains relatively fragile.

PM2.5 concentration (X7) is the strongest negative driving factor, with its impact on the EE displaying the distinct clustered distribution. The negative impact gradually intensified from the Shanxi-Shaanxi boundary area and the eastern part of Gansu towards the periphery. In particular, areas such as Dongsheng District, Kangbashi District, and Dingbian County exhibit stronger suppressive effects. As a concentration area for resource-based cities, the phenomenon of resource curse and path dependence has become increasingly prominent. The development of energy-intensive heavy industries has caused severe air pollution, which hinders the ecological environment.

The intensity of agricultural fertilizer application (X8) has both positive and negative impacts on the EE, exhibiting clear "northwest-southeast" disparity. The positive impact areas are mainly distributed in the northern part of Shaanxi, southern Inner Mongolia, and eastern Gansu. The negative impact areas are primarily found in most regions of Shanxi, central Shaanxi, and the Shaanxi-Gansu-Ningxia boundary areas. As a major agricultural production region, the Middle Yellow River Basin has a large proportion of ecosystem services provided by cropland. The positive impact suggests that appropriate levels of agricultural fertilizer can effectively increase crop yields and enhance supply services. The negative impact indicates that overuse of fertilizers is prevalent in the region.

The forest coverage rate (X9) is the strongest positive driver, with clear north–south spatial differentiation. The positive impact areas are concentrated in northern Henan, central Shaanxi, and southern Shanxi, with the intensity of impact decreasing from south to north. The higher the forest coverage, the stronger the ecosystem service capacity. The layered spatial distribution reflects differences in ecological baseline conditions and ecological engineering across regions. Forest coverage has a stronger promoting effect in the southern areas with better socio-economic development. In contrast, the northern areas, benefiting from the Three-North Shelterbelt Forest Program, already have a certain ecological baseline, so the impact is relatively smaller.

Market activity (X10) shows a clear east–west spatial difference in its impact on the EE. Positive effects are mainly distributed across most of Shanxi, northern Henan, and central Shaanxi, while negative effects exhibit a layered structure. The relationship between market activity and the ecological environment is subtle and intangible. The unreasonable consumption structure and consumption patterns can exacerbate resource depletion and ecological pressure.

The impact of per capita import and export volume (X11) on the EE shows the clustered distribution. Positive effects are mainly observed in the western regions of Gansu and Ningxia, central and western Shaanxi, and northern Henan. Negative effects are primarily seen in most of Shanxi, with the impact increasing from south to north. This indicates that in these regions, current import and export trade does not fully align with the demands of green development.

The number of students in compulsory education (X12) primarily exerts positive effect on the EE. The positive effect forms an axis from "northwest to southeast," with the impact decreasing from the center outward. The negative effect coefficients are very small, indicating that the negative impact on the ecological environment is not obvious. Education can promote individuals’ awareness of environmental issues, shape environmental values, and ultimately lead to changes in environmental behaviors.

## Discussion

### Exploring the spatiotemporal relationships between EE and HQED

The Middle Yellow River Basin, as the area of soil erosion and resource concentration, is critical target for the governance of the Yellow River Basin. This study takes the county level as the basic scale, which offers advantages in the research of the coupling between EE and HQED. The county level is the smallest scale for policy implementation, and socio-economic factors at scales smaller than the county level do not show considerable spatial heterogeneity^48^. The county scale avoids the averaging effect of larger scales such as urban agglomerations, provinces, or cities, which may overlook local differences, while also being more practical for regional development planning and implementation compared to grid-based scales^49^. Therefore, this study focuses on the 226 counties in the Middle Yellow River Basin, serving as a supplement and extension to existing research^50^.

In this study, substantial spatiotemporal differences in the EE and HQED are observed in the Middle Yellow River Basin. Regarding the EE, previous studies often calculated the ESV using the same value equivalent scale and equivalence factors, neglecting the regional differences. Additionally, factors such as price inflation can cause deviations in value accounting^51^, and the structure and functions of ecosystems exhibit clear spatial heterogeneity^52^. Therefore, using different provincial grain production levels, combined with the CPI and local NPP, can help refine the measurement of ESV in different regions^53^. The results indicate that the ecosystem service value in the Middle Yellow River Basin exhibits a trend of first decreasing and then increasing, which is corroborated by the finding of Zhang et al.^54^. The ecosystem services provided by cropland, forest, and grassland make up a large portion, aligning with the conclusion of Zhou et al.^55^. Programs such as the construction of key protective forests, conversion of cropland to forest and grassland, and soil conservation have had positive impact on improving the EE. At the same time, the dimensions of HQED have shown steady growth, with green development becoming the dominant dimension and innovation development showing the largest increase. Consistent with the findings of Zhang et al.^56^, the counties near provincial capitals and central cities are predominantly characterized by high levels, with obvious radiating effect. Additionally, there is spatial mismatch between the EE and HQED, similar to finding from studies in the Yangtze River Midstream^57^. This suggests that large river basins face similar challenges in coordinating ecological and economic development.

The coupling coordination degree between the EE and HQED in the Middle Yellow River Basin shows an upward trend, indicating that the new development philosophy and the priority of ecological development have been well implemented in socio-economic practices. However, some counties still exhibit serious and slight unbalances. In previous coupling coordination studies, it was often assumed that both systems held equal weight, overlooking the guiding role of government decisions. Therefore, by applying the strategy of main functional zoning and assigning weights to each county, we can more clearly evaluate the coupling coordination degree of each region. Before 2015, the coupling coordination degree showed no considerable changes, but after 2015, the number of balanced counties increased (by 65%). This is mainly due to the unification of ecological civilization and economic society, which improved the levels of EE and HQED between 2015 and 2020. Moreover, by examining the relative development degree, we can better identify the lagging types of specific counties, providing reference for regional development directions. For counties lagging in EE, such as Xi’an, the government should strengthen local ecological environment protection and restoration based on the integration of culture and tourism. For counties lagging in HQED, such as the Shanxi-Shaanxi boundary areas, the government should focus on promoting economic development based on ecological protection and low-carbon development, offering support through ecological compensation. Furthermore, the results of spatial autocorrelation indicate the positive spatial effect in the coupling coordination degree of the Middle Yellow River Basin. However, the spatial agglomeration areas did not considerably increase over time, which is inconsistent with the research on the Yangtze River Delta urban agglomeration^58^. This is mainly due to the fact that the Yangtze River Economic Belt has gradually formed a complete river basin system, while the counties in the Yellow River Basin are still constrained by provincial administrative boundaries and have not formed a complete scale effect within the basin. Therefore, the collaborative governance capacity of the Middle Yellow River Basin still needs to be further enhanced.

### Driving mechanism of HQED on EE

In the analysis of the driving mechanism by which high-quality economic development impacts the ecological environment, this study employs the VIF, RF, and GTWR models. Compared to the use of solely the Geographic Detector model or correlation analysis, this research accounts for the potential collinearity issues among driving factors, providing an intuitive reflection of each factor’s contribution to the EE^59^. The enhancement of EE quality is instrumental in supporting HQED, while HQED acts as the driving force for the improvement of EE quality^60^. Previous studies have mainly focused on the evaluation of coupling relationship between ecological protection and high-quality development in the Yellow River Basin or on external influencing factors, neglecting the internal driving mechanism between the two. This study, guided by the new development philosophy, further evaluates the impact of HQED on the EE, promoting the improvement of environmental quality from the source through HQED.

The driving factors indicate that forest coverage rate, PM2.5 concentration, agricultural fertilizer application intensity, and market activity contribute over 30% to the EE, making them key driving factors. Among them, forest coverage rate is the strongest positive driving factor, which is consistent with the finding of Qing et al.^61^. In areas with urbanization and dense populations, such as northern Henan and central Shaanxi, the positive impact of forest coverage rate on the EE is more obvious. PM2.5 concentration is the strongest negative driving factor, which aligns with the result of Ouyang et al.^62^. In particular, regions such as Dongsheng District, where energy industries and heavy chemical industries are concentrated, have considerable negative impact on the ecological environment. Although human activities inevitably impact the EE, their effects are not always negative^63^. The increase in the number of students in compulsory education helps raise environmental awareness, thus having positive effect on the EE. Factors such as industrial structure, urban–rural disparity, and market activity both promote and suppress the EE. These factors, as socio-economic development progresses, also exhibit an inverted U-shaped relationship between urbanization and the ecological environment^64^. In the early stages of socio-economic development, ecosystems are more vulnerable to disturbances; however, as economic quality improves and population quality increases, the EE will gradually improve. Additionally, the impact of agricultural fertilizer on the ecological environment requires further study. In major grain-producing areas, places like Dingbian County in the north have positive impact, while areas like Fuping County in the south show negative impact. Appropriate fertilizer usage helps increase grain yield, thereby improving food production services^65^, while excessive use of fertilizer results in suppressive effect.

### Zoning optimization strategies

**Fig. 12:**
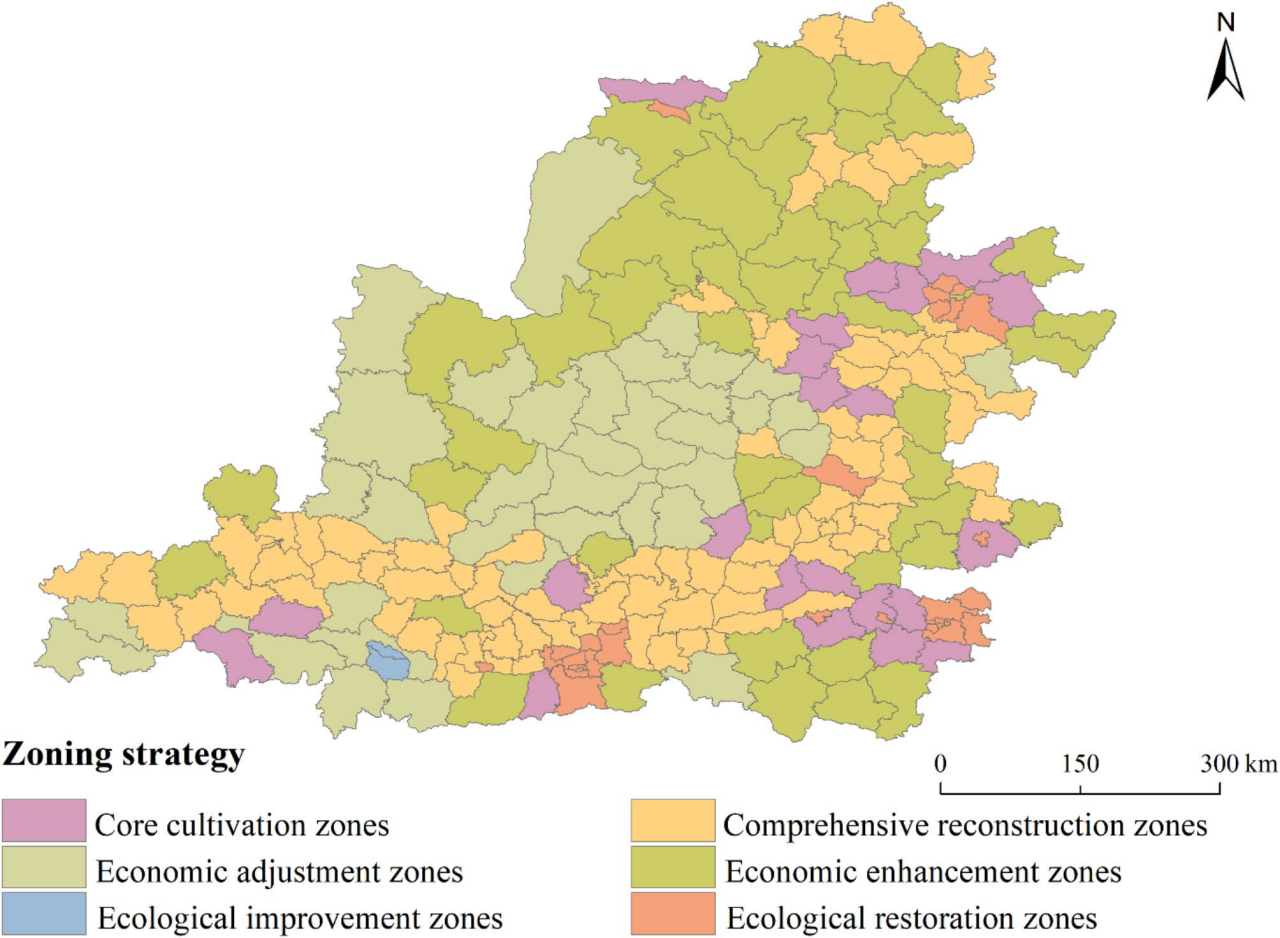
Zoning Management Strategies for EE and HQED in the Middle Yellow River Basin.

Based on the above content, combining coupling coordination types and driving mechanism, the zoning strategy for the EE and HQED in the Middle Yellow River Basin is proposed (Fig. 12). The coupling coordination characteristics of EE and HQED can be classified into four types: low-level harmony, high-level harmony, high-quality economic development lagging, and ecological environment lagging. We have identified the strongest driving factors within different counties as the dominant factors. Based on the direction of the dominant driving factors, the lagging type counties are further subdivided, resulting in six types across the 226 counties in the Middle Yellow River Basin.

Comprehensive reconstruction zones exhibit low levels of both EE and HQED. These regions require integrated management of the ecological environment and socio-economic systems, optimization of the industrial structure, and facilitation of the transition of traditional high-energy-consuming industries towards green industries. Eco-friendly industries, such as ecological agriculture and eco-tourism, should be cultivated to promote the coordinated development of the economy and the environment. Vegetation restoration projects should also be implemented to enhance the regional ecological foundation.

Core cultivation zones have high levels of both EE and HQED. It is essential to maintain the existing ecological environment quality while enhancing the stability and sustainability of economic development in these regions. Enhance the supervision and regulation of ecological protection, and establish a stringent ecological protection system. Promote cooperation and coordinated development among regions, leverage the radiating and leading role of core areas, and achieve sustainable regional economic development.

Economic adjustment zones are characterized by lagging HQED, with dominant driving factors exerting the suppressive effect on the EE. The government should adjust the development model, cultivate new economic growth points, and provide support through ecological compensation. Accelerate the formation of new types of industrial entities and advance the transition from old to new growth drivers to lay the foundation for the development of green industries.

Economic enhancement zones have lagging HQED, but the dominant driving factors have positive impact on the EE. These regions should focus on improving socio-economic development based on ecological protection and low-carbon development, with the emphasis on nurturing potential leading industries. It is crucial to actively develop green industries, nurture clean energy technologies, and enhance the overall development level of regional economies.

Ecological improvement zones have the lagging EE, and the dominant driving factors have suppressive effect on the EE. These areas should implement strict ecological protection measures, optimize land use structure, and improve ecological benefits. It is important to adhere to ecological red lines and enhance the region’s ecosystem service capacity. Optimize land-use structure to mitigate the ecological damage caused by irrational land development activities.

Ecological restoration zones have the lagging EE, but the dominant driving factors positively influence the EE. These areas are mostly centered around major cities, with dense populations, limited vegetation types, and relatively uniform land use types. Therefore, urban landscape patterns should be appropriately adjusted, with an emphasis on strengthening the connectivity of ecological corridors and landscape patches. Promote low-carbon lifestyles to facilitate the restoration and sustainable development of urban ecosystems.

### Limitations and future research

This study has the following limitations. Firstly, the ecosystem service value of construction land needs to be further clarified. Secondly, the thresholds of each driving factor still need to be explored. Lastly, due to data availability constraints and inherent model limitations, our findings may fail to capture critical short-term fluctuations. Therefore, future studies should conduct long-term time series analysis integrated with dynamic adjustment mechanisms for GTWR weighting functions, enabling more accurate investigation of the spatiotemporal interrelationships between EE and HQED.

## Conclusions

Based on the national strategic priorities of ecological protection and high-quality development in the Yellow River Basin, this study explores the relationship between the EE and HQED in the Middle Yellow River Basin, aiming to achieve unified accounting and evaluation of both the ecosystem and socio-economic systems. This research focuses on 226 counties in the Middle Yellow River Basin, analyzing the spatiotemporal differentiation and coupling coordination characteristics of the EE and HQED from 2010 to 2020. By employing RF models and GTWR models, the study investigates the driving mechanism of HQED on the EE. The finding not only expand the understanding of county-level ecological environments but also offer a new research perspective on the driving role of high-quality economic development. The main conclusions are as follows:

1. During the study period, the EE showed the trend of decline followed by an increase, while HQED displayed the steady upward trend. In terms of EE, the ESV provided by regulation services was the largest (411.21 × 10⁹ yuan in 2020), with forests contributing the most to the region’s ESV (46%). Spatially, counties with low ecological levels were mainly distributed in the eastern and southern areas, while high-level counties were primarily located in the northwestern regions. In terms of HQED, green development emerged as the dominant dimension, accounting for 25% of the total in 2020. Innovation development showed the largest growth, with an increase of 64% from 2015 to 2020. The spatial distribution followed the clear "southeast high, northwest low" pattern, with urban agglomerations and central cities exerting radiation effect. There exists the notable spatial misalignment between EE and HQED in the Middle Yellow River Basin.
2. The coupling coordination degree between EE and HQED showed an overall upward trend, but there are still many counties with unbalanced development, indicating that the coordinated development remains insufficient. Prior to 2015, there was no obvious change, but after 2015, the number of balanced counties increased considerably by 65%. Spatially, the distribution exhibited the pattern of "high in the southeast and northwest, low in the middle." In terms of spatial correlation, there was clear "high-high" and "low-low" clustering, demonstrating the strong positive spatial correlation. From the perspective of relative development, counties with low-level harmony continued to decrease, while counties with high-level harmony increased. Among the antagonistic counties, those with high-quality economic development lagging outnumbered those with ecological environment lagging.
3. Forest coverage rate, PM2.5 concentration, agricultural fertilizer application intensity, and market activity contribute over 30% to the ecological environment, making them key drivers. Among these, forest coverage rate is the strongest positive factor, while PM2.5 concentration is the strongest negative factor. In terms of spatial distribution, the regression coefficients for per capita GDP, forest coverage rate, and the intensity of agricultural fertilizer application exhibit the north–south differentiation pattern. The regression coefficients for industrial structure, urban–rural disparity, market activity, and per capita total import and export volume show the east–west distribution pattern. The regression coefficients for PM2.5 concentration are distributed in a clustered pattern. The regression coefficients for the number of students in compulsory education are distributed along a "northwest-southeast" axis. Based on the coupling coordination types and dominant driving factors, the study area is divided into six categories of regions, and corresponding development strategies are proposed.

## Data Availability

The datasets generated during and/or analysed during the current study are available from the corresponding author on reasonable request.
